# Tonics

**Published:** 1888-06-16

**Authors:** 


### THE DOCTOR'S PULPIT.

### TONICS.

MANY people think it is the easiest thing in the world to prescribe a tonic, and so they will go for one to the chemist, or take a friend's, or try Mother Seigel. The words "tone" and "tonic" are so wide, and include so many different ideas, conditions, and substances, that it is not surprising they should sometimes lead the unthinking into confusion. There are many words in every language which possess a very broad and inclusive significance. Take for example the word "builder" in our own. A man may be a house builder, or a ship builder, or a coach builder, or a "jerry" builder. But it is obvious that he who builds ships may be quite incompetent to build houses, and that the coach builder can have no experience in the construction of ships. The "jerry builder" is a dangerous person, and ought not to be allowed to build anything at all except for his own occupation. It is plain, then, that a tonic, being not one thing for one condition, but many things for many conditions, cannot be selected without discrimination by anybody and everybody who happens to feel the need of it.

A tonic may be required for the enrichment of the blood, in which case a particular kind of drug is necessary; or the nervous system may need toning up, and then a remedy of a totally different character may be demanded; or the stomach and the alimentary system generally may be "flat, stale, and unprofitable," calling probably for a line of treatment entirely antipodean to either of the former. These are not fancy pictures or exaggerated statements, but the veriest commonplaces of the consulting-room. What would be the result of setting a house builder to construct ships? Among immediate consequences a large waste of public money would be conspicuous, whilst among the more remote a foreign invasion might be anticipated. The probability is that he would not be able to produce anything the least resembling a ship; still less would he make his vessel a swift sailer, capable of carrying weight according to her tonnage. And yet the house builder, knowing something of the elements of construction in general, would be more likely to set about his shipbuilding intelligently than the amateur prescriber of tonics.

The illustrations already used may help us a little farther towards a clearly-defined position in a matter of daily practical importance. Given an intelligent ship builder on the Tyne, will he be able, by means of epistolary correspondence, to teach a house builder on the Clyde to construct useful seagoing ships? It seems to us that he will not. In like manner it is not to be expected that a newspaper writer sitting in an office in London or elsewhere can teach men and women in remote towns and country villages what is the best tonic to be taken at a given time. It is true a patient may describe his symptoms, but in the majority of cases his description will be entirely at variance with the facts. The point of the whole matter lies in the adage, "Every man to his trade."

It may be asked: Why then write about tonics, if the only object of writing is to say, "When you are ill the best course to pursue is to seek competent medical advice"? There is the very best reason for writing in the fact that tonics are much abused, to the great injury of those who take them. When a man is dyspeptic from eating too much beef it would be very bad treatment to attempt to cure his dyspepsia by a course of stale mackerel. And yet the use of ill-selected tonics by incompetent persons is just as foolish as the treatment of dyspepsia by mackerel would be. People think they can play with tonics. They do not reflect that the tonics used now are of a totally different character from the herbs and simples of our grandmothers' days. If a decoction of broom is found to be serviceable, there is no reason why a cottager should not make it for herself and use it. If horehound tea promotes appetite and digestion, by all means let it be indulged in. No coroner's jury, so far as medical science knows, ever brought in a verdict of "Died from drinking horehound tea." But the case is very different when quinine, the various preparations of steel, strychnia, arsenic, digitalis, and other dangerous substances are the components of the effective tonic. There is then "death in the bottle," if it be wrongly compounded or taken unintelligently. He who plays with drugs like these plays with edged tools, whether he acts the part of prescriber or the still more foolish part of swallower.

We are not unable to sympathise with him who desires to save the doctor's fee. Our instinct is to avoid the man of law when possible, and we are well aware that the patient may feel the same towards the doctor as the client does towards the lawyer. Nevertheless, he who in this busy age spends two or three hours in delivering a message himself in order to save the cost of a sixpenny telegram, is not a sound economist. It is precisely the same with the man who, to save a fee, is his own lawyer or doctor. In the modern world, division of labour is one of the conditions of civilisation and advancement. A man devotes himself to one thing in order that he may do that one thing as well as it can be done. It is contrary to all experience and common sense to suppose that he who devotes himself exclusively to the science and art of physic does not know more about it than he who devotes nine hundred and ninety-nine parts of his time to some other calling and gives the thousandth part, and a totally untrained mind, to the study of physic in his own person. It is as true of tonics as of every other kind of medical treatment. If you make up your mind to take physic, take it under the direction of a physician.

